# Cervical spine Rosai–Dorfman disease: A case report and literature review

**DOI:** 10.1097/MD.0000000000035654

**Published:** 2023-10-27

**Authors:** Xianfeng Rao, Zhengwen Kang, Jianwei Chen, Chang Cai, Bo Wang, Qiusheng Zhang

**Affiliations:** a Department of Neurosurgery, Shenzhen Second People’s Hospital, Shenzhen University First Affiliated Hospital, Clinical College of Shantou University Medical College, Shenzhen, China; b Department of Pathology, Shenzhen Second People’s Hospital, Shenzhen University First Affiliated Hospital, Shenzhen, China.

**Keywords:** Rosai–Dorfman disease (RDD), spinal lamina, epidural, subdural, tumor

## Abstract

**Background:**

Rosai–Dorfman disease is a benign lymphatic tissue proliferative disease with slow growth and is rarely observed in the clinic. Most of these patients present clinically with enlarged lymph nodes. In patients with spinal extranodal Rosai–Dorfman disease, which is even rare than the disease of lymph nodes, patients may experience numbness and weakness in the extremities.

**Case presentation:**

We report a 32-year-old male patient with multi-segmental spinal Rosai–Dorfman disease. On admission, his left fingers had been numb for 2 months. Over a 2-month period, the limb numbness progressed from the left to the right hand. The patient underwent resection of the lesion and internal fixation of the C2–C7 spine. The postoperative outcomes were satisfied and no recurrence was observed at 1-year follow-up.

**Conclusion:**

Spinal Rosai–Dorfman disease is a relatively rare tumor of which the pathogenesis is still unclear, and most patients have no specific clinical manifestations. Characteristic imaging findings can indicate this disease, however, a definitive diagnosis still depends on a pathological examination. Currently, total surgical resection of the tumor is a relatively effective and preferred treatment.

## 1. Introduction

Rosai–Dorfman disease (RDD) is a benign, slow-growing tumor that is rarely observed in clinical practice. This kind of tumor can occur at different sites, but usually in lymph nodes. RDD mostly presents as subcutaneous, isolated facial lymph node enlargement and is usually confined to the lymph nodes. RDD originating outside of the lymph nodes is rare, and a primary origin in the spine is even rarer. Most of these patients present clinically with symptoms of compression of the corresponding segment of the spinal cord, such as pain and numbness in the extremities or trunk. The severity of most clinical manifestations is related to the location of the primary tumor and the size of the tumor.^[1–5]^ Here, we report a case of spinal RDD that affected epidural, dural and subarachnoid spaces, we discussed its presentation, clinical features, imaging manifestations and treatment modalities.

## 2. Case presentation

### 2.1. Clinical history

A 32-year-old man presented with numbness of the extremities for 2 months before admission to hospital. The patient began to experience numbness in his left fingers in March 2021 without any apparent cause, which gradually progressed to the palm, the right upper limb, and finally, the trunk. He also had unsteadiness in holding objects in both hands, difficulty in performing fine movements, and pain in the back of his neck. These symptoms were worse at rest and could be relieved by activity. The patient had no nausea, vomiting, chest sulking, shortness of breath, cough, sputum, or other discomforts. He denied a history of hereditary disease in his family. He was admitted to the Department of Neurosurgery of the First Affiliated Hospital of Shenzhen University because of worsening symptoms. A physical examination showed decreased shallow and deep sensations in the left upper limb and decreased shallow sensation in the left thoracoabdominal wall (T2–L2 level). Routine blood laboratory tests, such as a full blood count, and renal and liver function tests, were within the normal range.

### 2.2. Imaging examinations

**Figure 1.**
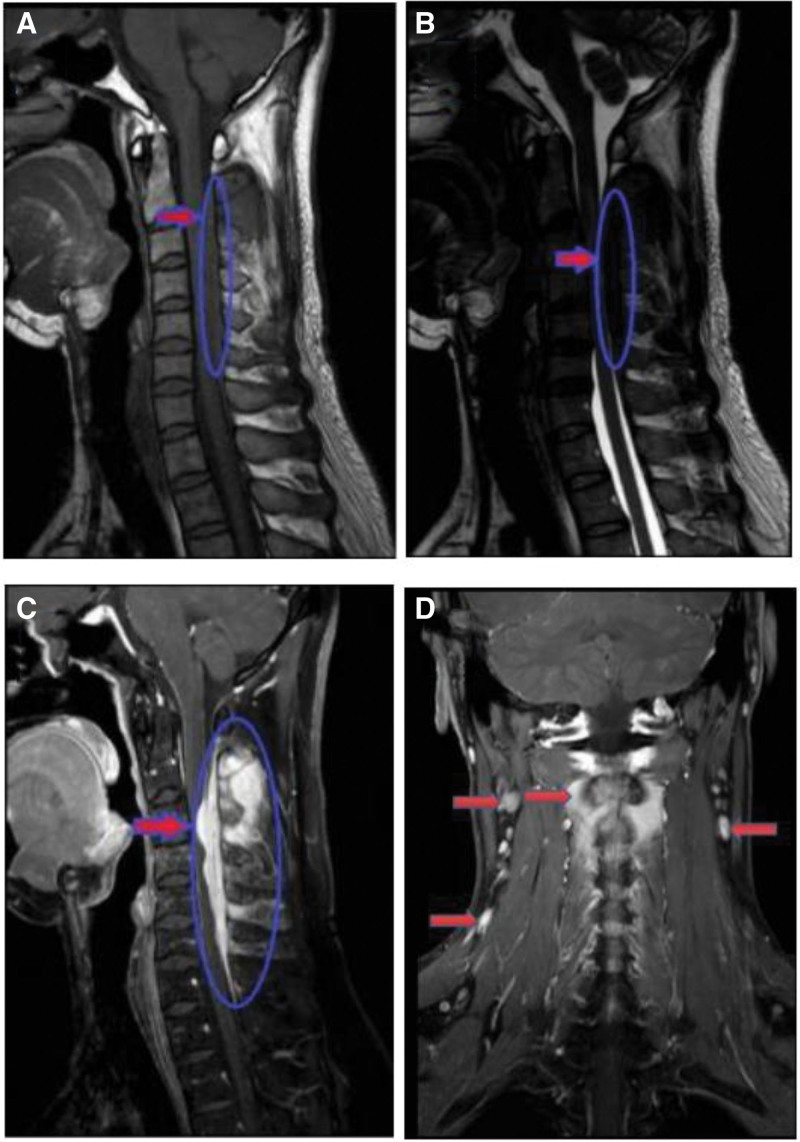
Contrast-enhanced magnetic resonance images of the spine. The lesion is hypointense on T1-weighted (A) and T2-weighted images (B). After intravenous administration of gadolinium, the tumor showed obvious peripheral enhancement and a tendency to compress the spinal cord anteriorly (C). Soft-issue-occupying lesions in the posterior neck with little cancellous bone destruction can be seen at the C2 and C3 levels. The lymph nodes in the neck are enlarged on both sides, and some lymph nodes are slightly enlarged (D).

Magnetic resonance imaging (MRI) of the spine showed that the mass was 45 × 23 × 46 mm in size. There was hypointensity on T1-weighted imaging (T1WI) and hypo- to isointensity on T2WI of an intradural extramedullary tumor at the C2–C5 level. After intravenous administration of gadolinium, the mass showed obvious enhancement and a tendency to compress the spinal cord anteriorly, without bone and soft tissue destruction. The bone signal of the C2–C5 spinous process was abnormal, the number of cervical lymph nodes was increased, and some lymph nodes were slightly enlarged. The diagnosis of potential eosinophilic granuloma at the C2–C5 level was made before surgery on the basis of the above-mentioned imaging findings (Fig. 1).

### 2.3. Surgical findings and pathological examination results

**Figure 2.**
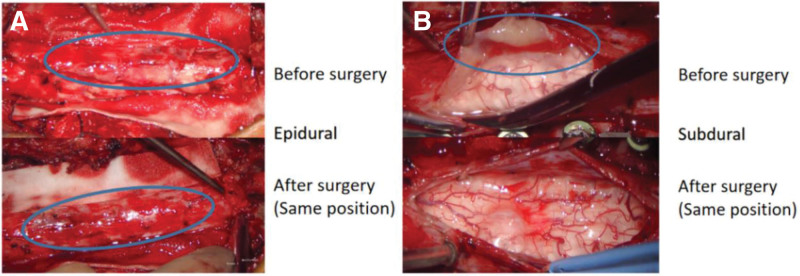
Two photographs from the surgery. The tumor was completely removed, including the epidural (A) and subdural (B) parts.

**Figure 3.**
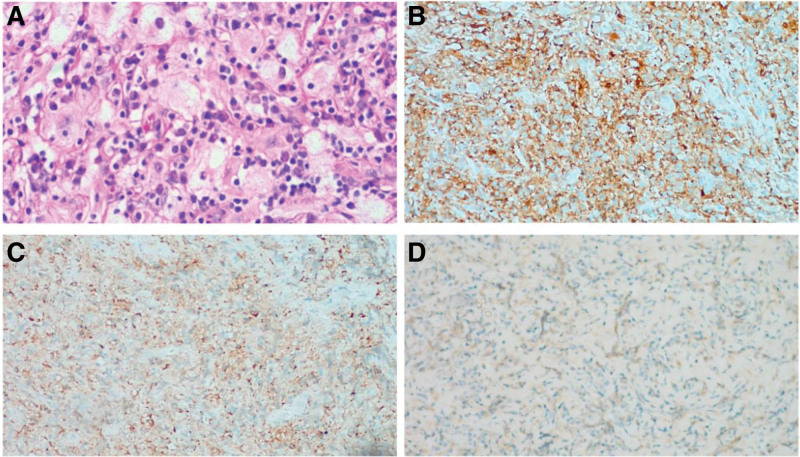
Histopathological images. The lesion comprises a large number of foam-like histiocytes, phagocytic lymphocytes, and infiltration of plasma cells and neutrophils. (A) Hematoxylin and eosin staining (magnification, ×40). The tumor cells are positive for S100 (B) and CD68, (C), but negative for CD1α (D) (magnification, ×100).

The patient was placed in the prone position with upper head frame fixation (Mayfield). The posterior median line of the C2–C7 spinous process was precisely positioned as the surgical incision by combining preoperative MRI, body surface markers, and a C-arm. A line for surgical incision was routinely drawn, disinfected, and toweled, and the posterior median approach was used. The skin and subcutaneous and deep fascia were dissected, and the muscles were separated on both sides along the spinous process and the vertebral plate on the lateral side. A microscopic examination revealed a yellowish, tough, blood-rich, irregular mass with poorly defined borders in the muscle adjacent to the C2–C6 spinous process. There was also partial destruction of the C2–C6 spinous process and vertebral plate bony structures. After microscopic total excision of the paravertebral tissue mass outside the spinal canal, the mass was sent for intraoperative frozen biopsy. The biopsy showed that the cells of the tumor were short and spindle-shaped, and there was no obvious heterogeneity. Additionally, some plasma cells and lymphocytes and neutrophil infiltration were observed around the tumor. The C2 and C7 muscle attachment points were stripped during the operation. In order to maintain patient postoperative cervical stability, the C2 pedicle screw and C3-C7 lateral mass screws were implantated. The epidural mass was totally removed from the spinal canal, and the dura mater was left intact. The tumor was carefully removed from the edge of the dura mater. After removal of the epidural mass, a distinct pressure mark was visible on the dura. When the C2–C7 dura was sliced using microscopy scissors, a yellowish subdural mass was immediately observed in the dorsal position under the extramedullary dura. The mass was carefully separated from the dura because it was not firmly attached and was totally removed. The dura mater was sutured tightly, and the C2–C7 segment was stabilized with plastic peptide rods before placing the first drainage tube, and the incision was closed. Throughout intraoperative neurophysiological monitoring, there were no abnormalities in movement or sensation (Fig. 2). The mass was 45 × 23 × 46 mm in size. A histopathological examination of the lesion showed characteristic findings. This examination showed that the lesion was yellow, soft, and well-circumscribed with an average blood supply and was located in the intraspinal epidural region. An immunohistochemical analysis showed that the tumor cells were positive for S-100 protein and CD68 and negative for CD1α. The final pathological diagnosis of the lesion was RDD (Fig. 3). There were not adverse and unanticipated events during the diagnosis and treatment process.

### 2.4. Postoperative outcome

**Figure 4.**
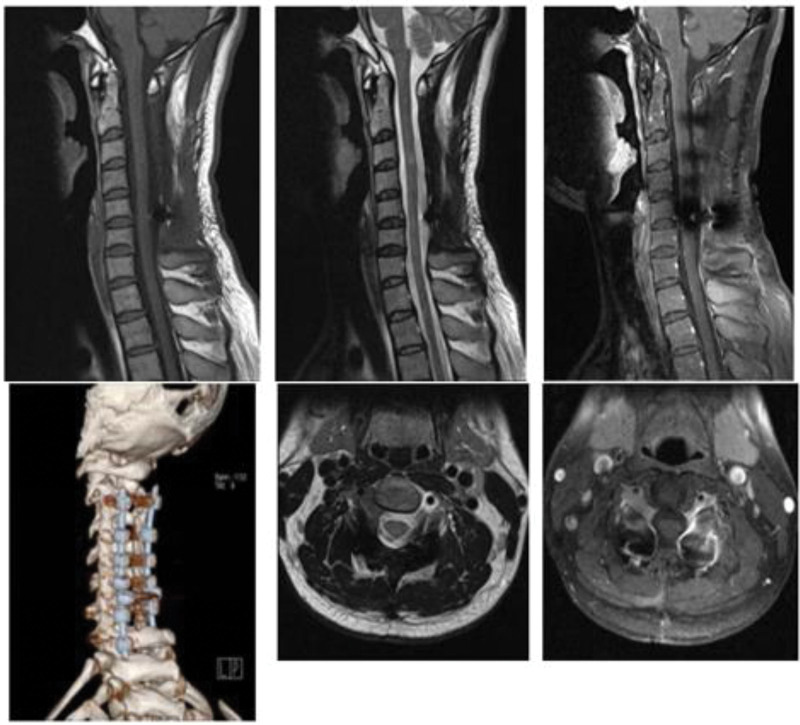
Postoperative magnetic resonance images 2 wk after surgery. The C2–C7 vertebral body and attachments are well retained with metal internal fixation. There is no loosening or fracture of the vertebral body and attachments, no occupation in the spinal canal, good morphology of the spinal cord without compression, and no abnormal enhancement on an enhancement scan.

**Figure 5.**
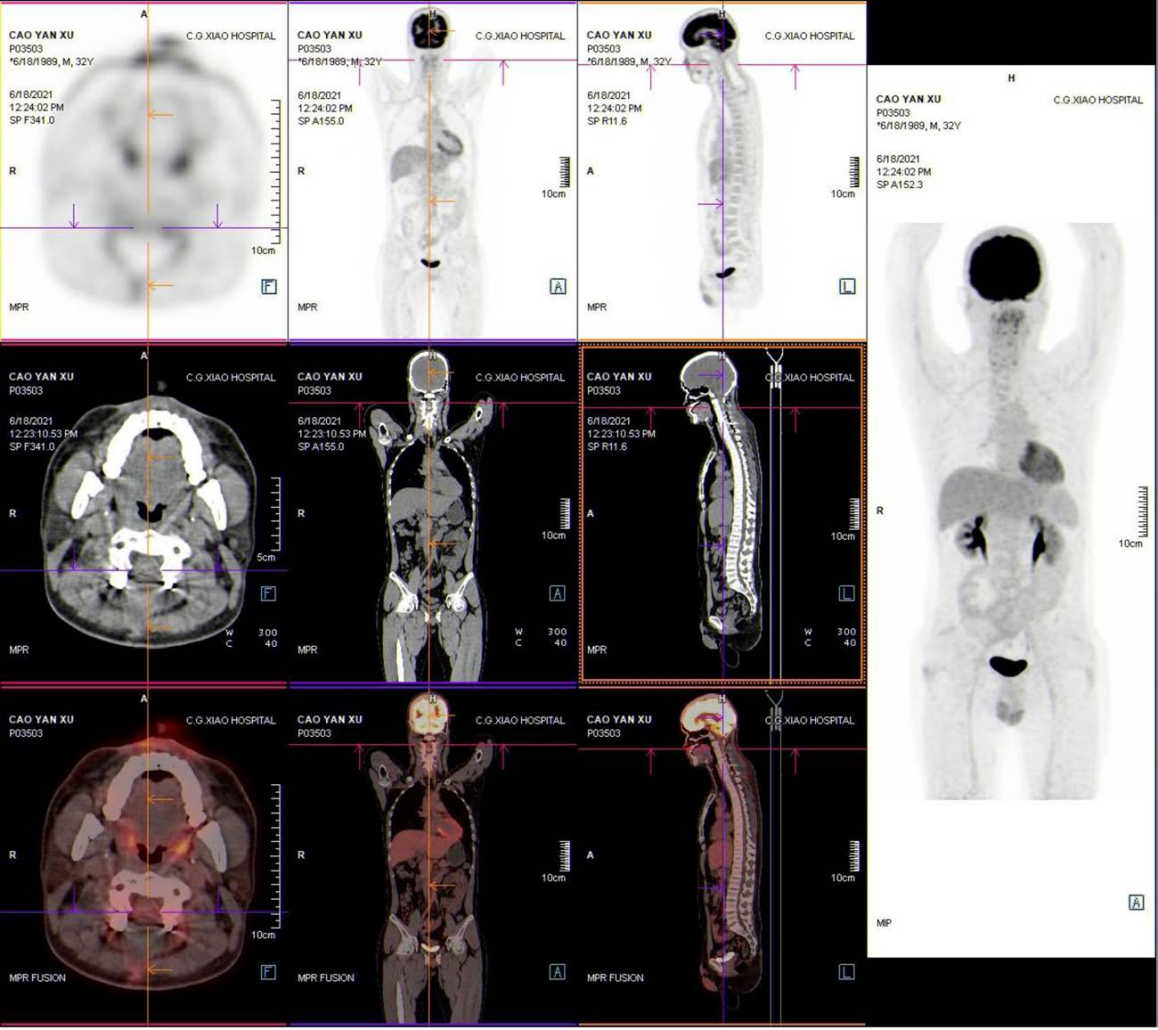
Postoperative cervical positron emission tomography–computed tomography 2 wk after surgery. The lesion did not involve other organs, and complete intraspinal surgical resection was performed.

**Figure 6.**
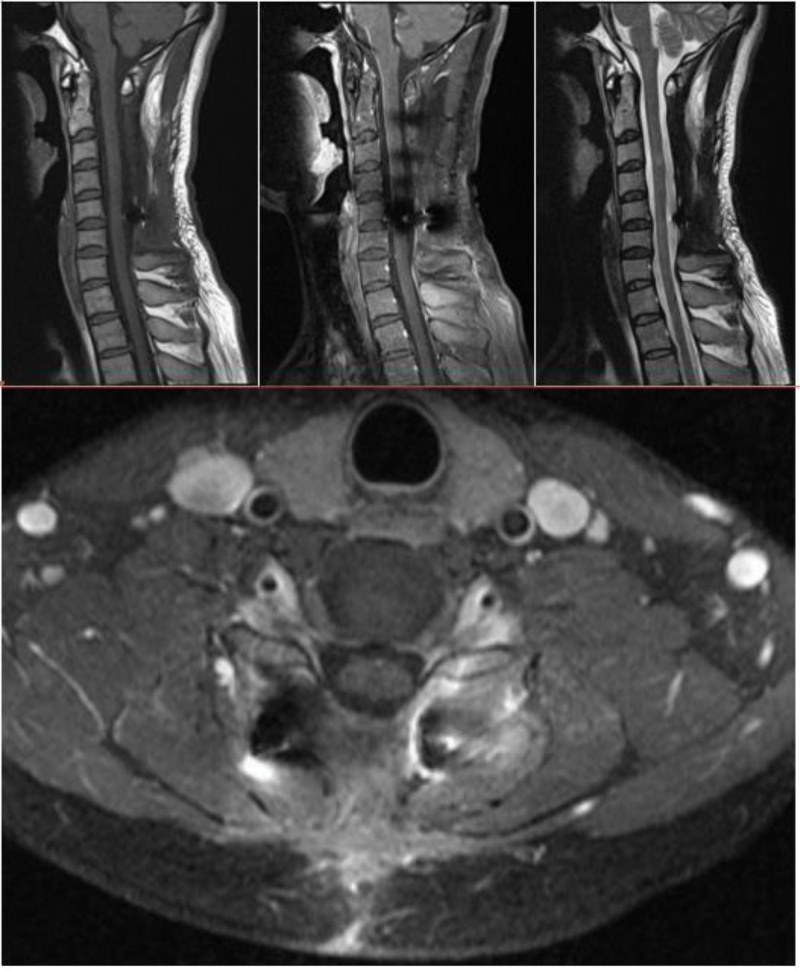
Second postoperative magnetic resonance imaging at 4 mo after surgery.

**Figure 7.**
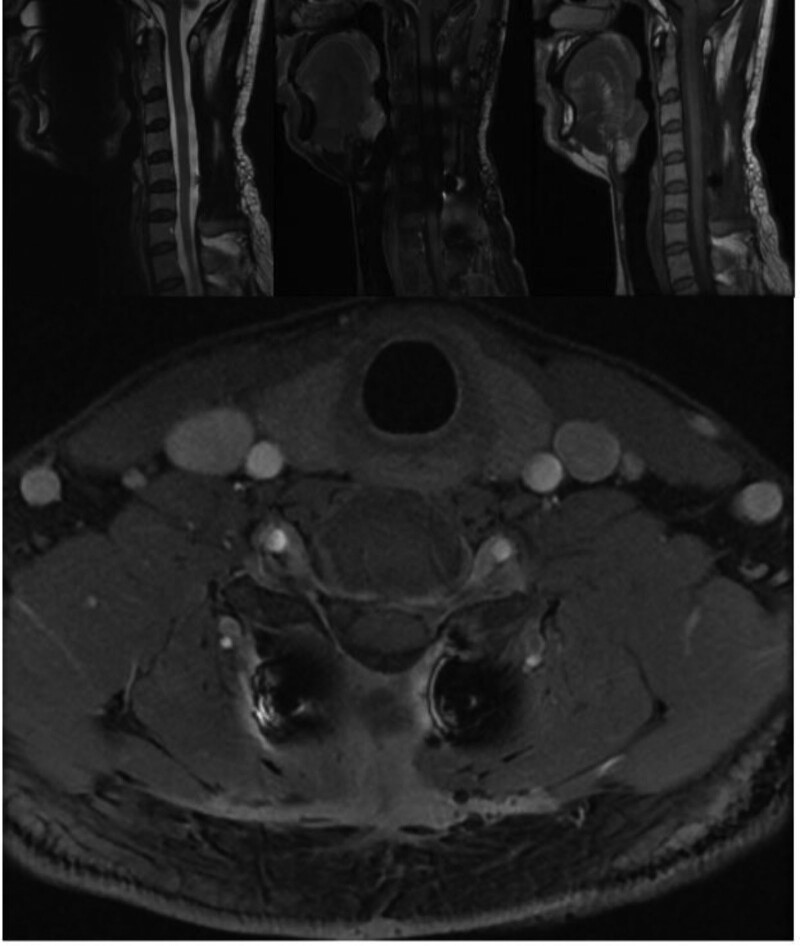
Third postoperative magnetic resonance imaging 10 mo after surgery.

**Figure 8.**
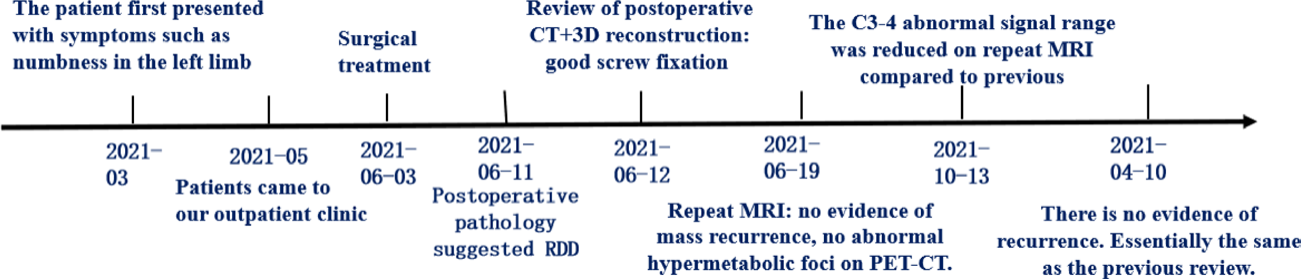
Timeline of the patient course.

The patient situation was stable with fully reserved neurological function and was discharged in a few days under a satisfactory condition after the operation. Postoperative MRI and positron emission tomography–computed tomography showed no obvious residual recurrence or metastasis 2 weeks after the surgery (Figs. 4 and 5). Postoperative MRI also showed no obvious residual or recurrence 4 and 10 months after the surgery (Figs. 6 and 7). A time line was also made to let the procedure of this case to become more clear (Fig. 8).

## 3. Discussion

RDD is a rare histiocytic disease originally described by Destombes in 1965 as giant lymphadenopathy with sinus histiocytosis.^[6]^ In 1987, Rosai and Dorfman further described RDD as non-Langerhans cell histiocytic hyperplasia The most common presentation of RDD in children and young adults is in the cervical lymph nodes.^[7]^ A large proportion of these patients also develop this disease with involvement of extranodal tissues, and the whole body may be involved.^[8]^ Extranodal involvement was recognized, and an analysis of the RDD registry was published in 1990 showing that 43% of cases were extranodal. The most common extranodal sites are the skin, upper respiratory tract, and bone.^[9]^ However, central nervous system involvement of RDD is still considered rare.^[10]^

Although the etiology of RDD is unclear, recent studies have shown that its occurrence is associated with a clonal origin. Therefore, RDD should be considered as a tumor rather than an inflammatory proliferative response.^[11]^ In 2016, RDD was classified by the World Health Organization as a hematopoietic and lymphoid tumor.^[12]^ A revised classification of histiocytosis published at the same time categorized various forms of RDD, from familial RDD to RDD associated with IgG4. RDD can be subdivided into lymph node (the most common), extranodal, and mixed types depending on the site of occurrence.^[13]^ In summary, the pathogenesis of RDD is unclear and needs to be confirmed by further research.

RDD is mostly cervical, massive, painless, peripheral, bilateral lymphadenopathy, and most tumors are painless with progressive enlargement.^[14]^ In our case, the patient had bilateral lymphadenopathy. When lesions occur in the spine, patients with RDD mainly show clinical manifestations of pinprick sensation, pain sensation, or hypesthesia and even paraparesis. The clinical manifestations of these patients are mostly related to the tumor growth site. Our patient presented with hypesthesia of the left limb.

MRI is the most commonly used imaging examination for RDD. However, there are also no specific MRI features indicative of RDD. MRI scans show that the tumor signals generally present as a homogeneous, isointense mass with clear borders and have obvious enhancement after contrast administration according to T1WI. However, on T2WI, these lesions appear to be heterogeneously hypo- to isointense. These findings are similar to those in our case. Pathological results are the gold standard for diagnosis, and the immunohistochemical characteristics of RDD include S100 (+), CD68 (+), and CD1α (−).^[9]^The imaging features of RDD are hypointensity on T1WI and T2WI and hyperintensity on T1WI with enhancement. Fifty percent of RDD cases present with multiple subcutaneous nodules that can be misdiagnosed as macula or papules.^[15,16]^

Because RDD is considered a benign disease, in cases without involvement of the central nervous system, conservative treatment is effective in 90% of patients. With regard to spinal RDD, surgical resection is still considered, because in addition to achieving complete resection and central nervous system decompression, mass for histological diagnosis can be obtained during surgery.^[17]^ There is limited evidence of the effectiveness of chemotherapy because of the rare incidence of RDD. Some studies have reported that tumor necrosis factor-α (thalidomide/rituximab) and other immunotherapies may be useful for systematic RDD.^[18]^

## 4. Conclusion

RDD is a benign proliferative disease, and early surgical excision provides the best prognosis for patients. The findings in our case show that surgical treatment alone is effective for treating cervical spine extranodal RDD without the need for postoperative radiotherapy and chemotherapy. Even if the tumor involves the spinal canal, it can still be removed surgically. Based on our experience, when tumors involving subdural region, in order to reach total resection, we suggest cutting the dura even if there is a high risk of postoperative leakage of cerebral spinal fluid, therefore, close sutures should be preferred after surgery to effectively avoied leakage of cerebral spinal fluid. In lesions involving multiple spinal segments, intraoperative internal fixation can be performed, which can reduce postoperative complications, such as spinal instability.

## Acknowledgments

We thank the patient and his family for participating in this case report.

## Author contributions

**Conceptualization:** Zhengwen Kang.

**Data curation:** Jianwei Chen, Chang Cai.

**Project administration:** Zhengwen Kang.

**Writing – original draft:** Xianfeng Rao, Jianwei Chen.

**Writing – review & editing:** Bo Wang, Qiusheng Zhang.
